# Surplus dietary isoleucine intake enhanced monounsaturated fatty acid synthesis and fat accumulation in skeletal muscle of finishing pigs

**DOI:** 10.1186/s40104-018-0306-5

**Published:** 2018-12-21

**Authors:** Yanhong Luo, Xin Zhang, Zhengpeng Zhu, Ning Jiao, Kai Qiu, Jingdong Yin

**Affiliations:** 1 State Key Lab of Animal Nutrition, College of Animal Science & Technology, China Agricultural University, Beijing, 100193 China; 2 Technology Research and Development Department, New Hope Liuhe Co. Ltd, Beijing, 100102 China

**Keywords:** Fatty acids synthesis, Intramuscular fat, Isoleucine, Monounsaturated fatty acids, Pigs, Skeletal muscle

## Abstract

**Background:**

Isoleucine (Ile) has been implicated in the regulation of energy homeostasis and adipogenesis. However, the impact of surplus dietary Ile intake on muscle lipogenesis remains unknown. The present study aimed to investigate the impact of dietary supplementation of extra-Ile on lipogenesis, fatty acid profile and lipid accumulation in skeletal muscle in finishing pigs.

**Methods:**

Forty-eight barrows with initial body weight of 77.0 ± 0.1 kg were allotted to one of two groups and fed diets containing 0.39%, 0.53% standardized ileal digestible (SID) Ile with six replicates per treatment and four pigs per replicate for 30 d.

**Results:**

Dietary Ile intake significantly improved the intramuscular fat (IMF) content and monounsaturated fatty acid (MUFA) concentration in the skeletal muscle (*P* < 0.05), and decreased the drip loss and shear force (*P* < 0.05) without influencing the growth performance of pigs (*P* > 0.05). Moreover, the phosphorylation of adenosine monophosphate activated protein kinase α (AMPKα) and acetyl coenzyme A carboxylase (ACC) proteins that monitor lipid metabolism were decreased in skeletal muscle of pigs offered extra-Ile diet (*P* < 0.05). The mRNA expression of adipose-specific genes adipocyte determination and differentiation factor 1 (*ADD1*), fatty acid synthase (*FAS*), and stearoyl-CoA desaturase (*SCD*) were upregulated and the activity of SCD was increased as well (*P* < 0.05).

**Conclusions:**

Surplus dietary Ile intake could increase IMF accumulation and MUFA synthesis in skeletal muscle through depressing the phosphorylation of AMPKα-ACC and stimulating the expression and activity of SCD, and increasing the capability of lipogenesis in skeletal muscle.

**Electronic supplementary material:**

The online version of this article (10.1186/s40104-018-0306-5) contains supplementary material, which is available to authorized users.

## Background

Pigs are the major source for human meat consumption and provide more than 40% of total meat production in the world [1]. The intramuscular fat (IMF) in pigs is closely associated with meat edible qualities such as flavor, juiciness, and tenderness as well as drip loss [2]. It includes phospholipids and triglycerides serving as the main forms of energy reserves and cholesterol [3]. The triglycerides are mainly stored in intramuscular adipocytes, and sarcoplasm in droplets, present themselves as the flecks and streaks of fat within the lean sections of meat [4]. Considering IMF content being a polygenic trait in livestock species, it is difficult to increase the IMF content genetically in a short-term. Therefore, it is more feasible to intervene nutritionally to increase IMF content and improve meat edible quality.

Amino acids not only serve as building blocks in protein synthesis [5, 6], but also act as nutritional signals, which play an important role in energy homeostasis and lipid metabolism [7, 8]. In pigs, the requirements for dietary amino acids have been well defined for obtaining optimal growth performance and fat-free lean meat production [9], while the impact of individual amino acid on meat quality of pigs remains unrevealed. It has been well accepted that branched-chain amino acids plays critical roles in the regulation of lipid metabolism [10–12]. However, the roles of individual branched-chain amino acids in regulation of energy metabolism and fat deposition in skeleton muscle have not been well elucidated. Isoleucine (Ile), one of branched-chain amino acids, plays an important role in energy homeostasis and lipogenesis [8]. It was observed that Ile deficiency significantly increasing fat mobilization in white adipose tissue and reducing body fat mass [8]. However, the effect of surplus dietary Ile intake on lipogenesis and fat accumulation remains unclear yet, especially in the skeletal muscle, which is closely associated with meat quality in pork production. Previous studies had shown that dietary supplementation of extra-Ile had no impact on the growth performance of pigs [13–15], but whether affect the meat quality and muscle lipogenesis remains unknown.

Therefore, the present study was carried out to study the effect of dietary supplementation of extra-Ile on lipogenesis, fatty acid profile and lipid accumulation in skeletal muscle in finishing pigs.

## Materials and methods

The experiment was performed at the Swine Nutrition Research Centre of the National Feed Engineering Technology Research Centre (Chengde, Hebei Province, China). All experimental procedures were approved by the Institutional Animal Care and Use Committee of China Agricultural University (ID: SKLAB-B-2010-003).

### Animals and diets

A total of 48 Duroc × Landrace × Large White crossbred barrows with an average body weight of 77.0 ± 0.1 kg (mean ± SD) were assigned into two dietary treatments in a complete randomized block design according to the initial body weight. There were six replicates (pens) per treatment and four pigs per pen (1.8 m × 2.1 m, 50% slatted floor). All the barrows were fed ad libitum and have a free access to clean drinking-water. The experiment last for 30 d. Experimental diets were formulated to meet NRC (2012) requirements for pigs of 75–100 kg body weight. Dietary Ile was set at the NRC (2012) recommended level (0.39% SID Ile) as the control diet, or exceeded the NRC (2012) level by 35% as the extra-Ile diet. The ingredient composition and nutrient level of the experimental diets were shown in Table 1 and Additional file 1: Table S1, respectively. Moreover, alanine was supplemented to maintain the same nitrogen level among the experimental diets.

**Table 1:** Ingredient composition of the experimental diets (%, as-fed)

Item	Treatments	
	Control	Extra-Ile
Ingredient		
Corn	85.14	85.08
Soybean meal (43.9% CP)	5.46	5.46
Wheat bran	3.00	3.00
Soybean oil	1.11	1.11
Limestone	0.95	0.95
Dicalcium phosphate	0.62	0.62
Salt	0.35	0.35
*L*-lysine HCl ^a^	0.55	0.55
*DL*-methionine ^a^	0.17	0.17
*L*-threonine ^a^	0.20	0.20
*L*-tryptophan ^a^	0.06	0.06
*L*-isoleucine ^b^	0.14	0.28
*L*-valine ^c^	0.13	0.13
*L*-histidine HCl ^c^	0.06	0.06
Phenylalanine ^c^	0.07	0.07
Alanine ^c^	0.27	0.19
Glutamic acid ^c^	0.55	0.55
Glycine ^c^	0.50	0.50
Vitamin-mineral premix^d^	0.67	0.67
Total	100.00	100.00

^a^ Provided by Dacheng Group, Changchun, China

^b^ Provided by Jiayuan Kang Company, Beijing, China

^c^ Provided by Health and Nutrition of Evonik Industries AG, Hanau, Germany

^d^ The premix provided the following per kg of diets: vitamin A 6,000 IU, vitamin D_3_ 2,400 IU, vitamin E 20 IU, vitamin K_3_ 2 mg, vitamin B_1_ 0.96 mg, vitamin B_2_ 4 mg, vitamin B_6_ 2 mg, vitamin B_12_ 0.012 mg, biotin 0.04 mg, folic acid 0.40 mg, pantothenic acid 11.2 mg, nicotinic acid 22 mg, choline chloride 80 mg, Cu (as copper sulfate) 120 mg, Fe (as ferrous sulfate) 76 mg, Mn (as manganese sulfate) 12 mg, Zn (as zinc sulfate) 76 mg, I (as potassium iodide) 0.24 mg, Se (as sodium selenite) 0.40 mg, phytase 100 mg

### Sample collection

At the end of the experiment, pigs close to the pen average body weight were fasted overnight. Blood samples of these pigs were collected from the anterior vena cava and serum was separated by centrifugation and stored at − 20 °C. After collection of blood, the pigs were killed by electrically stunned (250 V, 0.5A, for 5–6 s), and then exsanguinated and eviscerated. Samples of the *Longissimus dorsi* muscle (LDM) were rapidly separated from the right-side carcass at 10^th^ rib and then frozen in liquid nitrogen. Finally the muscle samples were kept at − 80 °C until for RNA and protein extraction, and enzyme activity measurement.

### Growth performance and carcass characteristics

Feed intake and body-weight were recorded on d 14 and d 30 to calculate the average daily feed intake (ADFI) and average daily gain (ADG). The ratio of ADG to ADFI (G/F) was used for feed conversion ratio.

After slaughter, hot carcass weight was recorded immediately, and dressing percentage was calculated dividing the hot carcass weight by the body weight. Backfat thickness was measured at the thickest shoulder, the last rib and the last lumbar vertebra according to the Chinese Guidelines on Performance Measurement Technology and Regulations for Pigs [16]. Loin eye height and width were measured at the center of the loin eye muscle at a right angle to get the maximum width (cm) and height (cm) at the last rib of the hung right side carcass, the loin eye area was calculated by the equation: loin eye area (cm^2^) = loin eye height (cm) × width (cm) × 0.7. Fat-free lean weight was estimated according to the equation fat-free lean weight = 0.95 × [7.231+ (carcass weight, pound) × 0.437] + (loin eye area, square inch) × 3 (NRC, 1998). The meat color score was scored according to the Official Color Standards (National Pork Producers Council, USA), a score of 1.0 is very pale, white and a score of 6.0 is dark purplish red. Drip loss was measured as described previously [17, 18]. Briefly, the meat close to 90 mg was hung in a plastic bag using string under the lid at 4 °C for 24 h, and the bag was kept out of contact with the meat. Drip loss was presented as a percentage of the amount of drip compared to the initial weight.

After slaughter, the 24 h-pH (pH _24h_) of the muscle was measured with a glass penetration pH electrode (pH-star, Matthäus, Germany). 24 h-meat color was measured three times using a tristimulus colorimeter (Minolta Chroma Meter Measuring Head CR-410 Minolta, Osaka, Japan) and obtained the ⊿L* (lightness), ⊿a* (redness) and ⊿b* (yellowness) values, and before measurement, the tristimulus colorimeter was calibrated using the white tile. Meat shear force was measured as described by Starkey et al. [19]. Each sample of the muscle was first cooking in a water bath at 70 °C for a period of 20 min. Ten cylindrical samples (1.0 in diameter) were obtained from cut the pork parallel to the fiber orientation, and measured by cut the sample vertically to the myofiber axis using a digital display muscle tenderness meter (C-LM3B, Tenovo, Harbin, China), speed 300 mm/min. The average for the 10 subsamples was recorded.

### Biochemical index assay

The concentrations of serum lipids, including total cholesterol (TC), triglyceride, low density lipoprotein (LDL)-cholesterol, high density lipoprotein (HDL)-cholesterol and non-esterified fatty acid, were determined using an automatic biochemistry analyzer (BS-420, Beckman, USA), and the contents of serum glucose and insulin were measured by commercial kits (Nanjing Jiancheng Institute of Bioengineering, Jiangsu, China).

### Intramuscular fat content

About 100 g of the LDM harvested at the 10^th^ rib of the right side carcass was frozen at − 20 °C for muscle chemical analysis in duplicate. About 50 g of the LDM of each sample was cut into thin slices (2–3 mm) and weighted in aluminum boxes. The boxes were then put into a vacuum frozen dryer (Freezone 4.5™, Labconco Corp., Kansas City, MO, USA) to freeze dry for 72 h. Lyophilized muscle was subsequently crushed into powder and fat content was measured by Soxhlet petroleum-ether extraction (Budwi Extraction System B-11; Budwi, Lausanne, Switzerland) as previously described [20]. IMF content was then converted to the weight percentage of fresh muscle weight.

### Feed composition analysis

Crude protein, calcium, and phosphorus in the experimental diets were measured according to the AOAC [21] procedures. The dietary amino acids were measured using a High Performance Liquid Chromatography with an Automatic Amino Acid Analyzer (L-8800 Amino Acids Analyzer, Hitachi, Tokyo, Japan) as described by Liu et al. [22].

### Muscle fatty acid composition

The fatty acid profile of the skeletal muscle was analyzed as described previously [23]. Briefly, 150 mg lyophilized muscle sample was extracted using 4 mL chloracetyl methanol (chloracetyl:methanol = 1:10), and treated with 1 mL n-hexane and 1 mL internal standard FA solution (1 mg/mL C11:0). After vortexed for 1 min, samples were kept in a water bath at 75 °C for 2 h and then added 5 mL potassium carbonate solution (70 g/L). The mixtures were centrifuged at 900 r/min for 5 min. The supernatant was analyzed by the Gas Chromatography (HP 6890 series, Hewlett Packard, Avondale, PA, USA), using a DB-23 capillary column (122–2362; length 60 m, internal diameter 0.25 mm, film thickness 0.25 μm; Agilent Technologies Inc., Santa Clara, CA, USA) and a flame ionization detector. Finally, according to the percentage of lyophilized muscle in fresh tissue, the fatty acids content is converted to levels in fresh tissue.

### Western blot analysis

To explore the alteration of lipogenesis and lipid accumulation in skeletal muscle, the relative protein expression levels of phosphor-adenosine monophosphate-activated protein kinase α (p-AMPKα), AMPKα, phosphor-acetyl coenzyme A (p-ACC), ACC, peroxisome proliferator activated receptor-γ (PPARγ) and CAAT/enhancer binding protein α (C/EBPα) in the LDM were determined by western blot analysis using the protein samples from all individual animals (*n* = 6) as described previously [24]. Briefly, equal amounts of samples (50 μg of protein), together with a pre-stained protein ladder (Thermo Fisher, Rockford, IL, USA), were subjected to sodium dodecyl sulfate polyacrylamide gel electrophoresis electrophoresis with glyceraldehyde 3-phosphate dehydrogenase (GAPDH) being run as a loading control. Subsequently, proteins were electro-transferred onto a polyvinylidene difluoride membrane and then blocked in 5% (*w*/*v*) bovine serum albumin at room temperature for 1 h. The membranes were incubated at 4 °C overnight against corresponding primary antibodies, including goat GAPDH, rabbit anti-phosphor-AMPKα (Thr^172^) and AMPKα, rabbit anti-phosphor-ACC (Ser^79^) and ACC, PPARγ and C/EBPα, all these antibodies were diluted to 1:1,000. All antibodies were purchased from Cell Signaling Technology (Beverly, MA, USA), except that GAPDH was purchased from HuaxingBio (Beijing, China). After three washes, the membranes were incubated with DyLight 800-labeled secondary antibodies. Finally, band signals were detected with the Odyssey Clx (LI-COR Biotechnology, Lincoln, NE, USA) and quantified using AlphaImager software (Alpha Innotech Corp., San Leandro, CA), then analyzed by Mann-Whitney U test using SAS software. All the results were normalized to GAPDH and data were expressed as the values relative to those for the 0.39% SID Ile treatment.

### Real-time PCR assay

Total RNA was extracted from the LDM using TRIzol reagent (R4115–02, Magen, Guangzhou, China) according to the manufacturer’s instructions. The concentration and quality of RNA were detected using a NanoDrop ND-1000 spectrophotometer (Thermo Scientific, Waltham, MA, USA). The total RNA was reverse transcribed into cDNA using the First Strand cDNA Synthesis Kit (CW0741A; CW Biotech Inc., Beijing, China). The relative expression of the genes was quantified by real-time qPCR (qTOWER 2.2 Real-Time PCR, Analytik Jena AG, Germany) with Takara real-time PCR Kit (RR096A; Takara Bio Inc., Tokyo, Japan). GAPDH gene was used to normalize the expression of target genes according to the formula 2^-ΔΔCt^, where ΔΔCt = (Ct_target_–Ct_GAPDH_) treatment − (Ct_target_ − Ct_GAPDH_) control. All the samples were measured in triplicate. The primers sequences for genes were presented in Table 2.

**Table 2:** Primers used for real-time qPCR

Gene^a^	Primers	Primer sequences (5′→3′)	Size, bp	Tm, °C	Accession No.
*ADD1*	Forward	GCGACGGTGCCTCTGGTAGT	218	60	XM_021066226.1
	Reverse	CGCAAGACGGCGGATTTA			
*FABP4*	Forward	TGGAAACTTGTCTCCAGTG	147	54	NM_001002817.1
	Reverse	GGTACTTTCTGATCTAATGGTG			
*FAS*	Forward	GCCTAACTCCTCGCTGCAAT	195	60	NM_001099930.1
	Reverse	TCCTTGGAACCGTCTGTGTTC			
*HSL*	Forward	TCGGAGTGAACGGATTTG	195	60	–
	Reverse	TCCTCCTTGGTGCTAATCTCGT			
*LPL*	Forward	CTCGTGCTCAGATGCCCTAC	148	60	NM_214286.1
	Reverse	GGCAGGGTGAAAGGGATGTT			
*SCD*	Forward	GCCTACTATCTGCTGAGTGC	152	57	XM_021072070.1
	Reverse	TCTCGGGCCCATTCATAAAC			
*GAPDH*	Forward	TCGGAGTGAACGGATTTG	219	60	NM_001206359.1
	Reverse	CCTGGAAGATGGTGATGG			

^a^*ADD1* Adipocyte determination and differentiation factor 1, *FABP4* Fatty acid binding protein 4, *FAS* Fatty acid synthase, *HSL* Hormone sensitive lipase, *LPL* Lipoprotein lipase, *SCD* Stearoyl-CoA desaturase, *GAPDH* Glyceraldehyde-3-phosphate dehydrogenase

### Activities of lipogenic enzymes in skeletal muscle

The enzyme-linked immuno sorbent assay, colorimetric method as described [25], and radioimmunoassay were used to determine the activity of fatty acid synthase (FAS), lipoprotein lipase (LPL) and stearoyl-CoA desaturase (SCD), respectively. The unit of LPL activity U/mg was defined as one micromole free fatty acid produced per milligram protein of tissue per hour, and the unit of SCD activity U/mg was defined as one nanomole oleic acid produced per milligram protein of tissue per minute. All the enzymes activities in LDM were measured by the commercial kits (Nanjing Jiancheng Institute of Bioengineering, Jiangsu, China) according to the manufacturer’s guidelines.

### Statistical analysis

Growth performance data were analyzed by ANOVA of SAS; other data were analyzed by unpaired t-test procedures of SAS (Version 9.3, SAS Institute, Cary, N.C.) with each animal as an experimental unit. Data were expressed with mean ± SEM values. *P* < 0.05 was considered to be significant different and 0.05 ≤ *P* < 0.10 was considered to have a trend.

## Results

### Growth performance and carcass characteristics

**Fig. 1:**
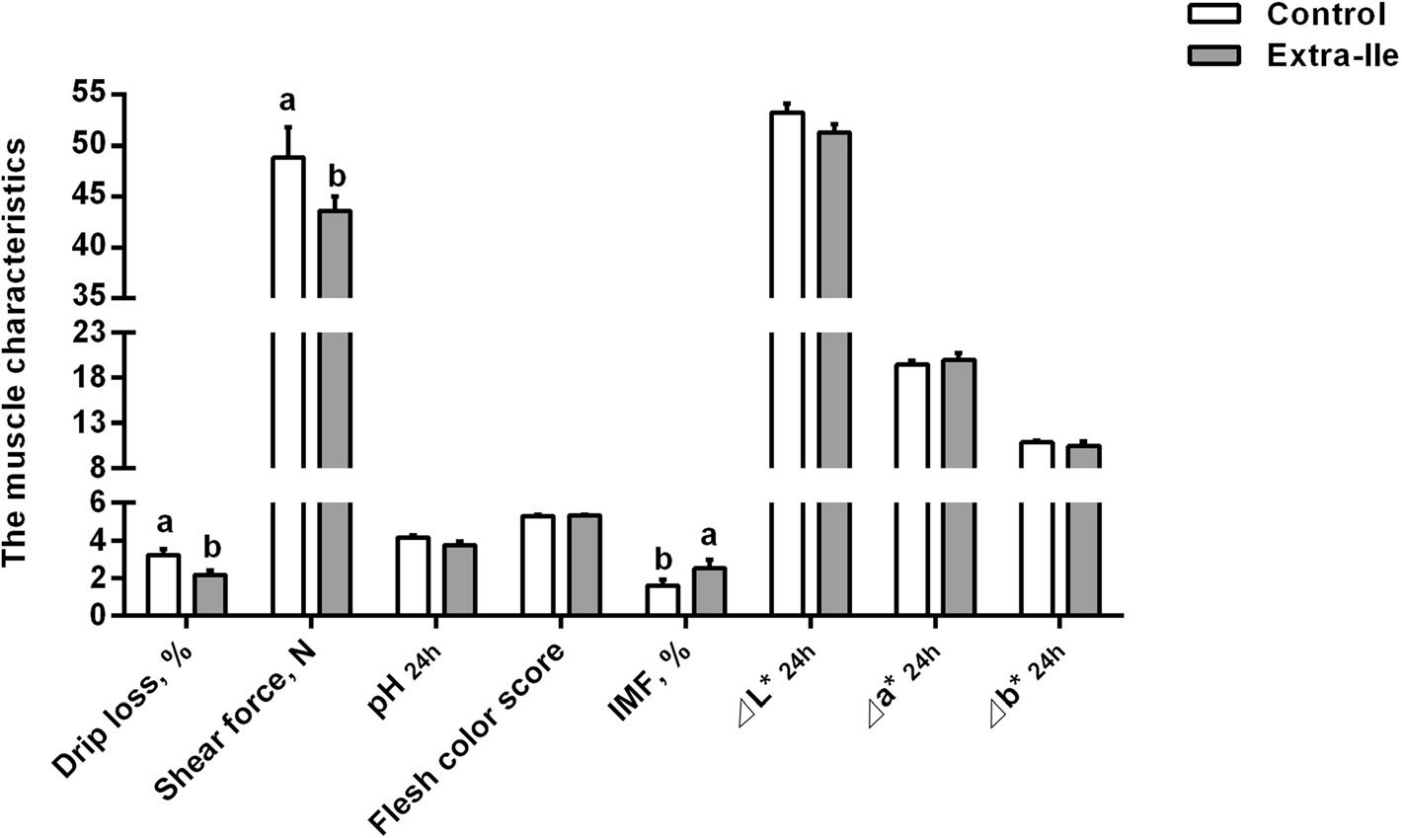
Effects of dietary supplementation of extra-Ile on the meat quality of finishing pigs. Data were represented as mean ± SEM (*n* = 6). IMF, intramuscular fat. ^a,b^ Values with different letters are significantly different (*P* < 0.05)

The growth performance of ADG, ADFI and G/F ratio were not affected by dietary supplementation of extra-Ile (*P* > 0.05) (Table 3). The carcass weight (*P* = 0.05) and the fat-free lean weight (*P* = 0.07) tended to be decreased by the extra-Ile diet, while the dressing percentage, loin eye area and backfat depth of the carcass were not influenced (*P* > 0.05). IMF content was significantly increased by dietary addition of extra-Ile (Fig. 1), and the drip loss and shear force of muscle decreased (*P* < 0.05) (Fig. 1), and the pH_24h_, flesh color score and ⊿L*_24h_, ⊿a*_24h_, ⊿b*_24h_ values were not influenced (*P* > 0.05). These data indicated that dietary inclusion of extra-Ile did not affect growth performance, but improve the meat quality by increase IMF and decrease drip loss and shear force.

**Table 3:** Effect of dietary supplementation of extra-Ile on growth performance and carcass characteristics of finishing pigs^a^

Item	Treatments		SEM	*P*-value
	Control	Extra-Ile		
Initial body weight, kg	77.0	77.0	0.1	0.99
Final body weight, kg	105.9	104.6	1.5	0.57
ADG, kg/d	0.92	0.89	0.04	0.69
ADFI, kg/d	2.84	2.78	0.10	0.71
G/F	0.32	0.32	0.01	0.56
Carcass weight, kg	86.80	83.50	0.92	0.05
Dressing percentage, %	76.31	74.59	0.69	0.14
Loin eye area^b^, cm^2^	50.99	46.70	1.69	0.13
Fat-free lean weight^c^, kg	46.55	44.15	0.75	0.07
Subcutaneous backfat depth, cm				
Shoulder fat thickness	3.68	3.33	0.23	0.33
The last rib fat thickness	1.68	1.64	0.10	0.76
Lumbosacral fat thickness	1.52	1.48	0.16	0.86
Average backfat depth	2.23	2.15	0.13	0.48

^a^ Values are means with pooled SEMs, *n* = 6. *ADG* Average daily gain, *ADFI* Average daily feed intake, *G/F* Gain to feed ratio

^b^ Loin eye area was measured at the last rib of the right side carcass, and calculated by the equation loin eye area (cm^2^) = loin eye height (cm) × width (cm) × 0.7

^c^ An estimated method according NRC (1998). Fat-free lean weight = 0.95 × [7.231 + (carcass weight, pound) × 0.437] + (loin eye area, square inch) × 3

### Serum metabolites

Serum metabolites can partly reflected the physiological functions and metabolic status of the body. Serum concentrations of TC, HDL-cholesterol and LDL-cholesterol (*P* < 0.05) were decreased by the extra-Ile diet, but serum glucose concentration (*P* < 0.05) was increased. The serum concentrations of triglyceride, non-esterified fatty acid and insulin were not influenced by the extra-Ile diet (*P* > 0.10) (Table 4). This indicated that surplus dietary Ile intake could alter lipid metabolism and decreased the serum contents of TC, HDL-cholesterol and LDL-cholesterol.

**Table 4:** Effects of dietary supplementation of extra-Ile on the serum lipids and glucose of finishing pigs^a^

Item	Treatments		SEM	*P*-value
	Control	Extra-Ile		
TC, mmol/L	3.20	2.79	0.06	0.01
Triglyceride, mmol/L	0.41	0.55	0.05	0.10
HDL-cholesterol, mmol/L	0.91	0.77	0.02	0.01
LDL-cholesterol, mmol/L	1.49	1.35	0.03	0.02
Non-esterified fatty acid, mmol/L	0.47	0.47	0.04	0.89
Glucose, mmol/L	5.41	5.91	0.10	0.02
Insulin, μIU/mL	15.23	15.00	0.54	0.78

^a^ Values are means with pooled SEMs, *n* = 6. *TC* Total cholesterol, *HDL* High density lipoprotein, *LDL* Low density lipoprotein

### Fatty acid profile in the LDM

As shown in Table 5, dietary supplementation of extra-Ile significantly increased the content of oleic acid C18:1n-9c and total monounsaturated fatty acid (MUFA), and tended to increase the concents of palmitoleic acid (C16:1) (*P* = 0.09) and eicosenoic acid (C20:1) (*P* = 0.08) in the skeletal muscle (Table 5). Among all these changes, C18:1n-9c, the largest proportion of fatty acid, was significantly increased by extra-Ile diet. This indicated that the supplementation of extra-Ile in the diet increased the IMF content may especially through increased the MUFA content in muscle. In addition, surplus dietary-Ile intake significantly decreased the ratio of *n*-6/*n*-3 polyunsaturated fatty acid (PUFA) (*P* < 0.05).

**Table 5:** Effect of dietary supplementation of extra-Ile on the fatty acids profile in the LDM of finishing pigs (mg/g, of fresh tissue)^a^

Item	Treatments		SEM	*P*-value
	Control	Extra-Ile		
Myristic acid (C14: 0)	0.67	0.74	0.03	0.14
Palmitic acid (C16: 0)	12.83	14.34	0.64	0.15
Stearic acid (C18: 0)	7.11	7.31	0.39	0.73
Heneicosanoic acid (C21: 0)	0.21	0.23	0.01	0.33
Tetracosanoic acid (C24: 0)	0.24	0.24	0.02	0.84
Palmitoleic acid (C16: 1)	1.64	2.01	0.13	0.09
Oleic acid (C18: 1n-9c)	20.55	24.43	0.95	0.03
Eicosenoic acid (C20: 1)	0.41	0.49	0.03	0.08
Linoleic acid (C18: 2n-6c)	5.25	4.12	0.54	0.20
Alpha-linolenic acid (C18: 3n-3)	0.15	0.17	0.01	0.16
Eicosatrienoic acid (C20: 3n-6)	0.19	0.21	0.02	0.57
Arachidonic acid (C20: 4n-6)	1.24	1.26	0.09	0.89
∑ SFA	21.66	23.63	1.05	0.24
∑ MUFA	22.79	27.12	1.09	0.04
∑ PUFA	7.05	5.98	0.66	0.31
∑ n-6 PUFA	6.75	5.66	0.64	0.28
∑ n-3 PUFA	0.30	0.32	0.02	0.44
n-6/n-3	22.69	17.52	0.96	0.01
PUFA/SFA	0.33	0.25	0.03	0.11
Total fatty acids	51.48	56.73	2.33	0.17

^a^ Values are means with pooled SEMs, *n* = 6. *SFA* Saturated fatty acid, *MUFA* Monounsaturated fatty acid, *PUFA* Polyunsaturated fatty acid

### Expression of adipose-specific genes in the LDM

To understand the underlying mechanism of extra-Ile intake inducing alteration of lipid accumulation in muscle, we examined the AMPK pathways and key genes related to lipid metabolism.

**Fig. 2:**
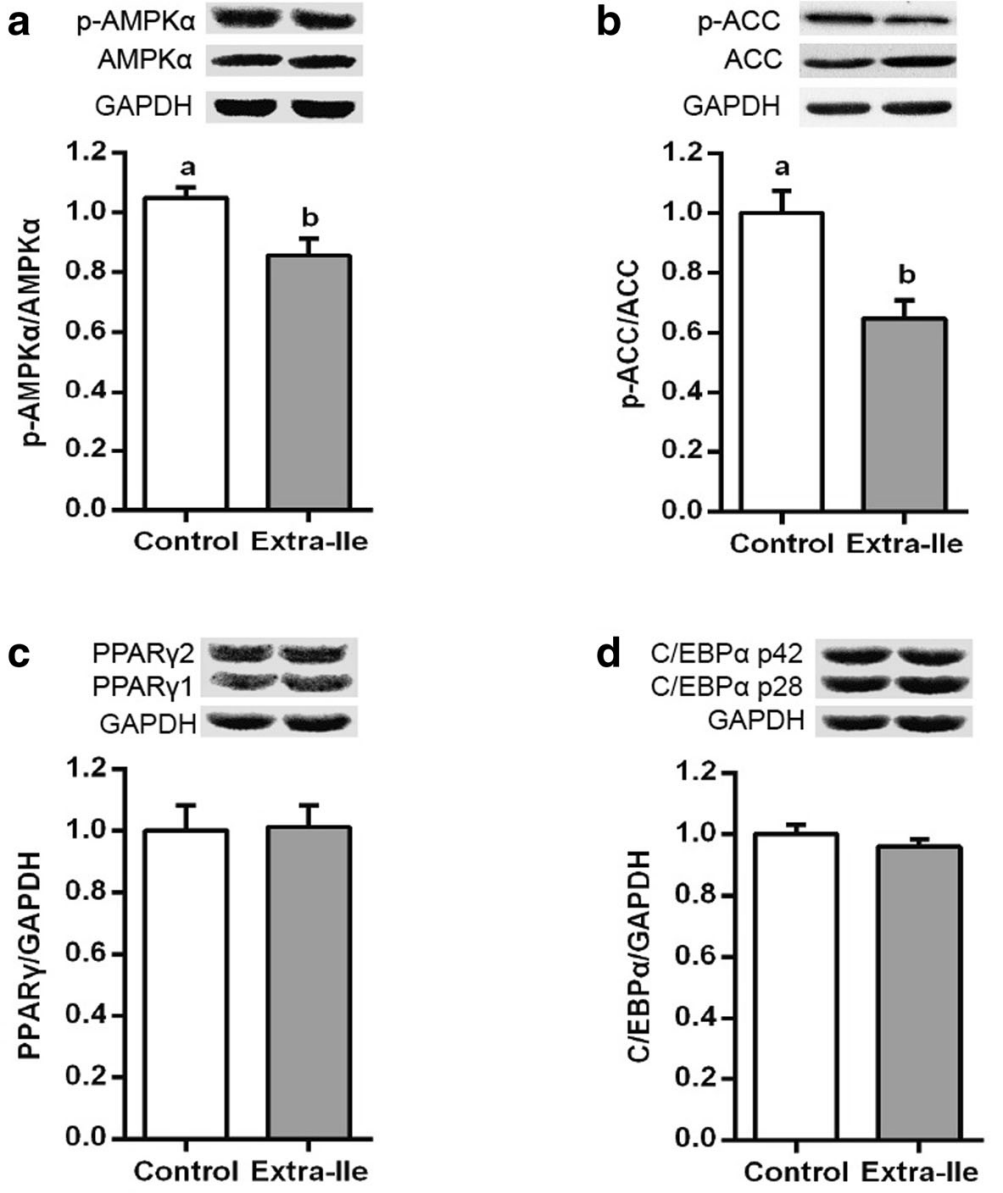
Western blot results reflect the effect of dietary supplementation of extra-Ile on the expression of AMPK signaling in the LDM of finishing pigs. Data were represented as mean ± SEM (*n* = 6). **a** The phosphorylation of AMPKα protein; (**b**) the phosphorylation of ACC protein; (**c**), the expression of PPARγ protein; (**d**), the expression of C/EBPα protein. AMPKα, adenosine-activated protein kinase α; ACC, acetyl coenzyme A carboxylase; C/EBPα, CCAAT/enhancer-binding protein α; GAPDH, glyceraldehyde 3-phosphate dehydrogenase. ^a,b^ Values with different letters are significantly different (*P* < 0.05)

As shown in Fig. 2, the phosphorylation of AMPKα and ACC protein expression were decreased in the skeletal muscle in pigs fed the extra-Ile diet (*P* < 0.05) (Fig. 2a and b). Meanwhile, protein expression of PPARγ and C/EBPα were not altered by the extra-Ile diet (Fig. 2c and d).

**Fig. 3:**
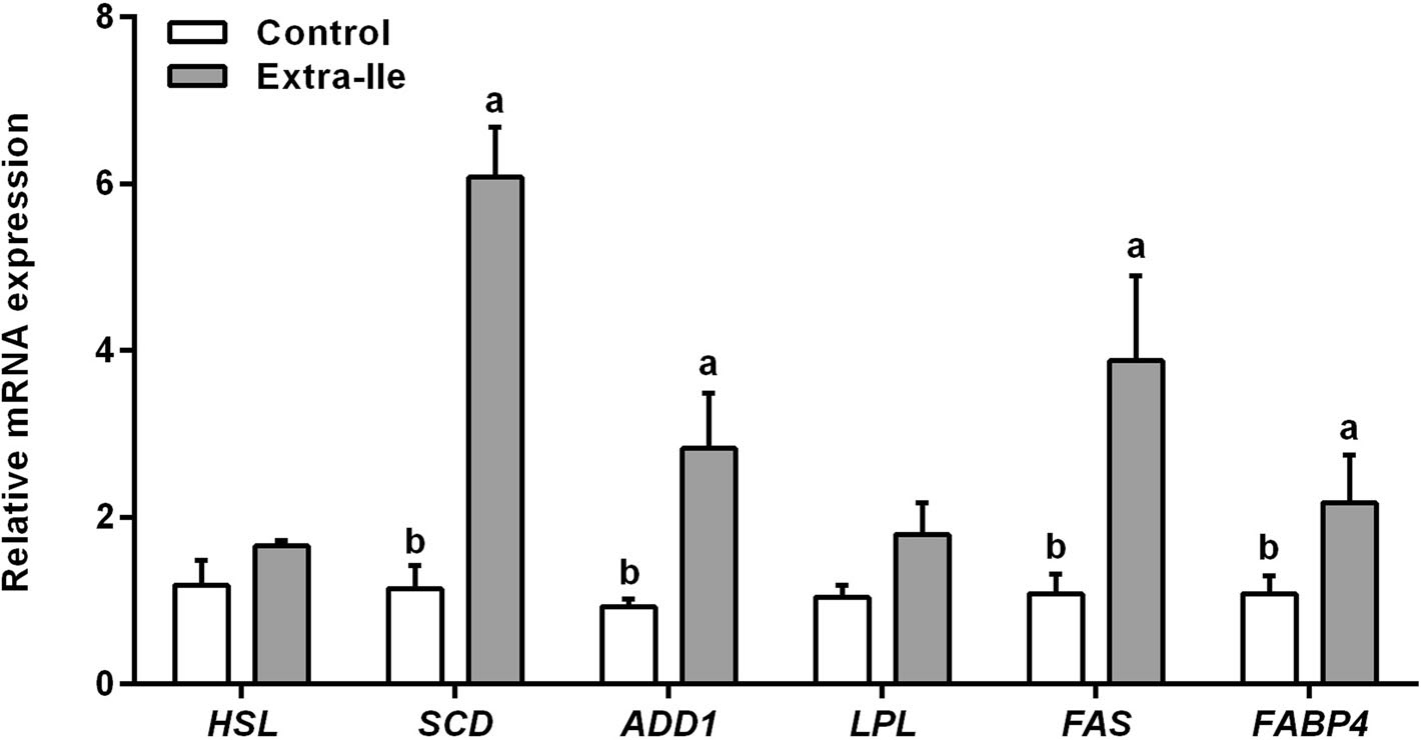
Effects of dietary supplementation of extra-Ile on mRNA expression of adipose-specific genes in the LDM of finishing pigs. Data were represented as mean ± SEM (*n* = 6). *HSL*, hormone sensitive lipase; *SCD*, stearoyl-CoA desaturase; *ADD1*, adipocyte determination and differentiation factor 1; *LPL*, lipoprotein lipase; *FAS*, fatty acid synthase; *FABP4*, fatty acid binding protein 4. ^a,b^ Values with different letters are significantly different (*P* < 0.05)

**Fig. 4:**
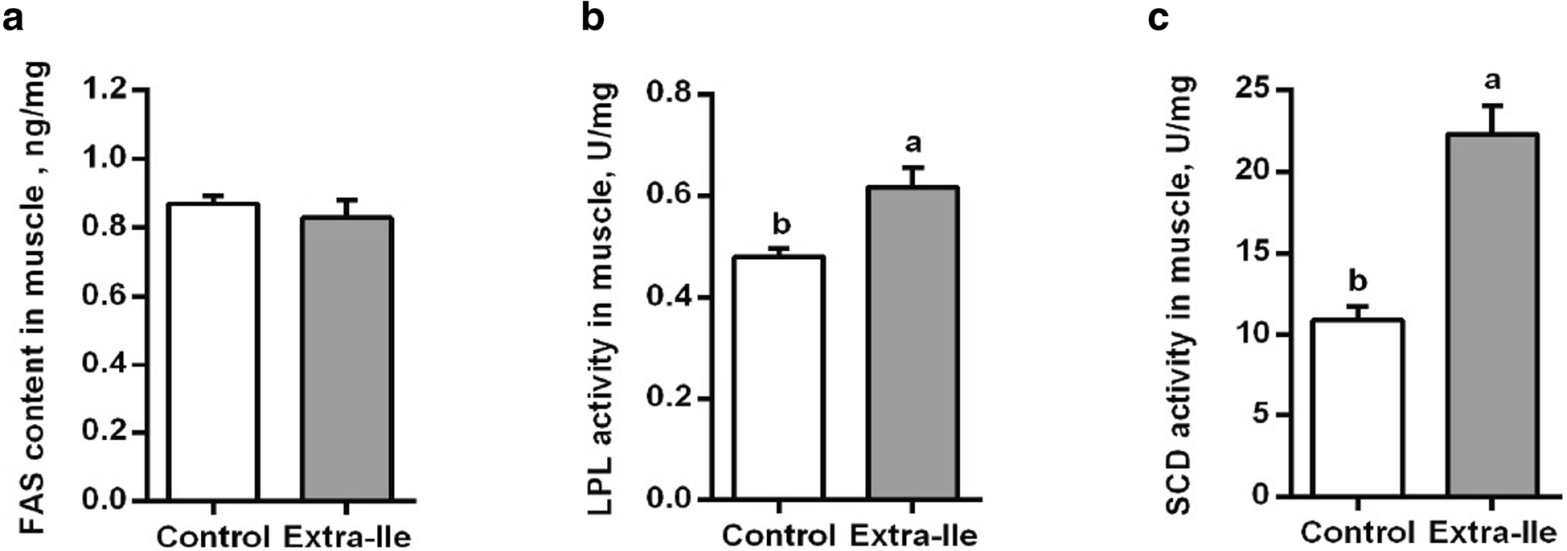
Effects of dietary supplementation of extra-Ile on the activity of enzymes related to lipogenesis in the LDM of finishing pigs. Data were represented as mean ± SEM (*n* = 6). **a** The activity of FAS; (**b**), the activity of LPL; (**c**), the activity of SCD. FAS, fatty acid synthase; LPL, lipoprotein lipase; SCD, stearoyl-CoA desaturase. ^a,b^ Values with different letters are significantly different (*P* < 0.05). The U/mg unit of LPL activity was defined as one micromole free fatty acid produced per milligram protein of tissue per hour, and the U/mg unit of SCD activity was defined as one nanomole oleic acid produced per milligram protein of tissue per minute

As shown in Fig. 3**,** the mRNA expression of adipocyte determination and differentiation factor 1 (*ADD1*), *SCD*, *FAS* and fatty acid binding protein 4 (*FABP4*) were increased in skeletal muscle of pigs fed the extra-Ile diet relative to the pigs on control diet (*P* < 0.05). Meanwhile, mRNA expression of hormone sensitive lipase (*HSL*) and *LPL* were not altered. Regarding the enzyme activity, LPL and SCD activities in the skeletal muscle were significantly increased (*P* < 0.05) in pigs offered the extra-Ile diet compared with the control (Fig. 4b and c), while FAS activity was not altered (*P* > 0.05) (Fig. 4a). These results showed that dietary supplementation of extra-Ile decreased the phosphorylation of AMPK, and also upregulate the expression of lipid synthesis related genes, especially increase the SCD activity which monitors the MUFA synthesis.

## Discussion

In the present study, we observed that the ingestion of a diet containing extra-Ile than the NRC by 35% did not affect the ADG, ADFI, and G/F of finishing pigs, which were consistent with previous study [14]. But the supplementation of extra-Ile tended to decreased the carcass weight and fat-free lean weight of finishing pigs, which was different with previous study that fat-free lean weight was not altered by supplying extra 20% Ile in a corn-blood cell diet. The discrepancy in terms of fat-free lean weight may be due to the different levels of Ile included in diets. The IMF is composed of extramyocellular lipids stored in adipocytes interspersed between fibers and intramyocellular lipid within myocytes [26–28]. It is well accepted as an important index of meat quality in livestock and greatly contributes to the meat eating acceptability [29–31]. In previous study, IMF content was improved in pigs offered the diet containing increased levels of Ile and valine while the total amount of branched-chain amino acids was kept constant [32]. In the present study, the IMF content was increased by dietary extra-Ile supplementation. This indicated that surplus dietary Ile intake may not alter the body weight, but change muscle composition, and such changes may be related to the alteration of muscle lipid metabolism induced by extra Ile.

The fatty acids profile of the fat components in muscle is associated with meat edible quality [2]. The saturated fatty acid (SFA) and MUFA correlate with meat flavor positive, while PUFA have a negative correlation [33]. Fatty acids in muscle mainly come from two sources: one is from lipogenesis and reflected by the varied proportion of SFA and MUFA in tissues; the other one is from diet, such as PUFA, and accumulates unchanged in the tissues [34]. Therefore, to a certain extent, the variation of skeletal muscle fatty acid profile reflects the balance between body lipogenesis and fatty acids absorption from food intake. In our study, surplus dietary Ile intake significantly increased the content of oleic acid (C18:1n-9c) and MUFA, and tended to increase the palmitoleic acid (C16:1) and eicosenoic acid (C20:1), and decreased n-6/n-3 PUFA in the skeletal muscle. These results indicated that the changes of the fatty acid profile in muscle resulted from dietary inclusion of extra-Ile could be relate to elevated MUFA synthesis in muscle.

**Fig. 5:**
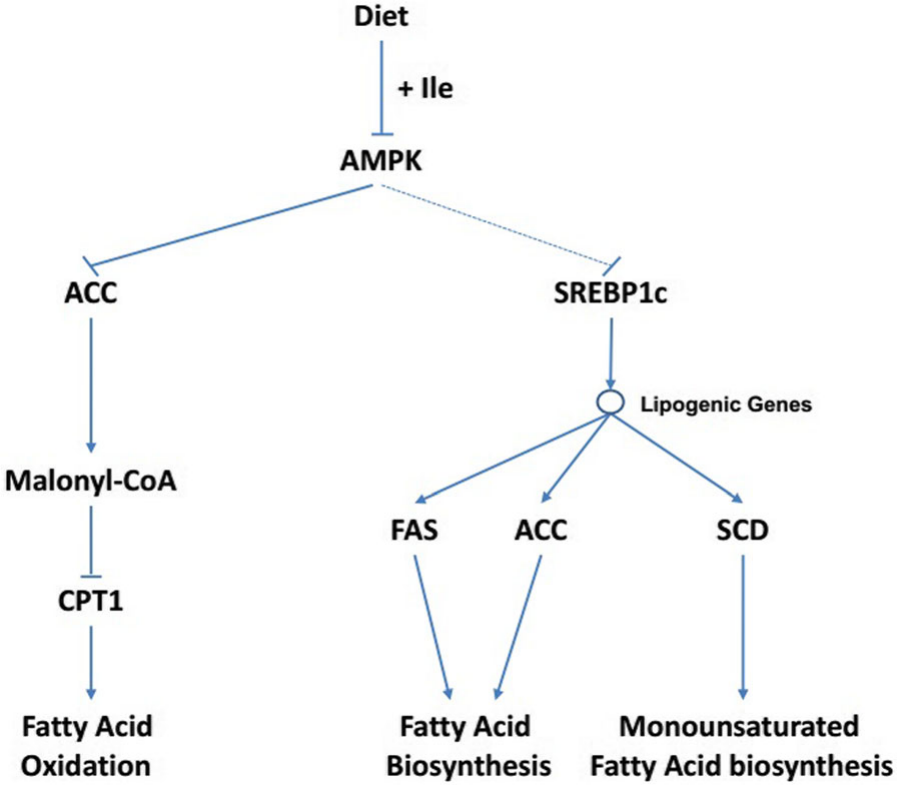
The potential mechanism that extra-Ile intake induced alteration of lipid metabolism in muscle through AMPK pathways. ACC, acetyl coenzyme A carboxylase; AMPK, adenosine-activated protein kinase; CPT1, carnitine palmitoyl transferase 1; FAS, fatty acid synthase; SCD, stearoyl-CoA desaturase; SREBP1c, sterol response element-binding protein 1c

It has been shown that branched-chain amino acids play an important role in adipogenesis [35]. So the alteration of IMF and MUFA content increase in muscle caused by surplus dietary Ile supply could be associated with the modification of key pathways that modulate muscle lipogenesis and lipid metabolism. AMPK is a key protein that regulates body energy metabolism and lipogenesis [36, 37]. ACC, a target gene of AMPKα, is a rate-limiting enzyme of lipogenesis, and depressed by its phosphorylation [38]. The phosphorylation of AMPK stimulates the phosphorylation of ACC, and depresses ACC activity, and subsequently decreases the synthesis of malonyl-CoA, which inhibits the carnitine palmitoyl transferase 1 activity, therefore increase the fatty acid oxidation; on the other hand, decreased activity of ACC retards the pivotal step of the fatty acid synthesis pathway [39]. In our study, extra Ile supply could decrease the phosphorylation of AMPKα and ACC protein, and consequently activated the ACC activity and improved the fatty acid synthesis (Fig. 5). This was evidenced by the increased contents of MUFA and IMF in the skeletal muscle of pigs offered extra-Ile diet. Moreover, the above results also demonstrated that extra Ile supply may affect the energy consumption by decrease the fatty acid oxidation (Fig. 5), and we also found the serum glucose was increased by surplus Ile, which meant that the utilization of glucose and energy may also decreased. Previous studies showed controversial results about the glucose and energy metabolism induced by BCAA. It had been shown that dietary leucine extra intake improves glucose metabolism and increase energy expenditure in mice maintained a high-fat diet [40], however, it had been demonstrated that Ile or leucine deprivation could decrease the serum glucose and increase fatty acid oxidation and energy expenditure [8, 41]. So, whether the increased serum glucose and decreased muscle fatty acid oxidation were due to extra Ile induced energy utilization or consumption decrease needs further research to confirmation.

Sterol response element-binding protein 1 (SREBP-1)/ADD1, acts as an important positive transcription factor in nutritional induction of the FAS promoter via the − 278/− 131 region [42]. In our study, compared to the control, the ingestion of the diet containing extra-Ile significantly increased the mRNA expression of *SREBP-1/ADD1* and *FAS*, and improved the IMF content in the skeletal muscle. However, FAS activity was not altered by extra-Ile diet in the skeletal muscle, which suggested that medium-term ingestion of extra-Ile diet only increased the potentiality of *FAS* just on the level of mRNA expression, rather than increasing its activity directly. LPL activity was increased in the skeletal muscle of pigs offered extra-Ile diet compared with the control, which implied that extra-Ile enhanced lipogenesis in the skeletal muscle was also associated with increased transfer of fatty acids from the circulation.

Stearoyl-CoA desaturase (SCD), a rate-limiting enzyme in the biosynthesis of MUFA, can catalyze the conversion of palmitate and stearate to their corresponding unsaturated fatty acids (C16:1, C18:1) that can be further used for the synthesis of cholesterol and phospholipid [43]. In the present study, that extra-Ile intake increased both the expression and activity of SCD, which was confirmed by the increased MUFA content in skeletal muscle. FABP4, a member of the fatty acid binding protein family, is involved in lipogenesis [44], and positively correlated with IMF contents [45]. In our study, the mRNA expression of *FABP4* was increased when pigs were offered extra-Ile diet. Additionally, dietary Ile treatment did not influence the mRNA expression of *HSL* which means extra-Ile intake have no impact on the muscle lipolysis.

Obviously, lipogenesis is an integrated process involving of many adipose-specific enzymes, such as ACC, SCD, FAS, LPL, and etc. The present study implied that the increased expression and activity of SCD by extra-Ile intake may play a major role in promoting MUFA synthesis and IMF accumulation in the skeletal muscle. Moreover, it had been shown that dietary MUFA supplementation can decrease the serum total and LDL-cholesterol concentrations and lower the serum lipid concentrations [46]. So we speculated that the decreased serum TC, HDL-cholesterol and LDL-cholesterol of pigs offered Ile diet may be associated with the increase of MUFA synthesis.

## Conclusion

Combining with increased MUFA and IMF content, we effectively demonstrated that dietary supplementation of extra-Ile could enhance lipogenesis, MUFA synthesis and fat accumulation in skeletal muscle by depressing the phosphorylation of AMPKα-ACC, and stimulating SCD expression and activity.

## Additional file

Additional file 1: **Table S1.** Nutrient composition of the experimental diets (%, as-fed). (DOCX 17 kb)

## Abbreviations

ACC: Acetyl coenzyme A carboxylase
ADD1: Adipocyte determination and differentiation factor 1
ADFI: Average daily feed intake
ADG: Average daily gain
AMPKα: Adenosine monophosphate -activated protein kinase α
C/EBPα: CCAAT/enhancer-binding protein α
FABP4: Fatty acid binding protein 4
FAS: Fatty acid synthase
G/F: Gain to feed ratio
GAPDH: Glyceraldehyde 3-phosphate dehydrogenase
HDL: High density lipoprotein
HSL: Hormone sensitive lipase
Ile: Isoleucine
IMF: Intramuscular fat
LDL: Low density lipoprotein
LDM: *Longissimus dorsi* muscle
LPL: Lipoprotein lipase
MUFA: Monounsaturated fatty acid
PPARγ: Peroxisome proliferator activated receptor-γ
PUFA: Polyunsaturated fatty acid
SCD: Stearoyl-CoA desaturase
SFA: Saturated fatty acid
SID: Standardized ileal digestible
SREBP-1: Sterol response element-binding protein 1
TC: Total cholesterol
