# Supplementary material for: Surplus dietary isoleucine intake enhanced monounsaturated fatty acid synthesis and fat accumulation in skeletal muscle of finishing pigs
Source: J Anim Sci Biotechnol. 2018 Dec 21;9:88. doi: 10.1186/s40104-018-0306-5 (PMC6302484; doi:10.1186/s40104-018-0306-5)
Supplement: Supplementary file 1 — Table S1. Nutrient composition of the experimental diets (%, as-fed). (DOCX 17 kb) [file 40104_2018_306_MOESM1_ESM.docx]

**Additional file**

**Table S1** Nutrient composition of the experimental diets (%, as-fed)

| Item | Treatments, % | |
| --- | --- | --- |
|  | Control | Extra-Ile |
| Calculated composition |  |  |
| Digestible energy, kcal/kg ^1^ | 3,384 | 3,391 |
| Metabolizable energy, kcal/kg ^1^ | 3,310 | 3,317 |
| SID isoleucine ^2^ | 0.39 | 0.53 |
| SID leucine ^2^ | 1.00 | 1.00 |
| SID lysine ^2^ | 0.73 | 0.73 |
| SID methionine + cysteine ^2^ | 0.42 | 0.42 |
| SID threonine ^2^ | 0.46 | 0.46 |
| SID tryptophan ^2^ | 0.13 | 0.13 |
| SID valine ^2^ | 0.48 | 0.48 |
| Calcium | 0.52 | 0.52 |
| Total phosphorus | 0.37 | 0.37 |
| STTD phosphorus | 0.24 | 0.24 |
| Analyzed composition ^3^ |  |  |
| Crude protein | 11.93 | 12.05 |
| Total isoleucine | 0.45 | 0.58 |
| Total leucine | 0.92 | 0.92 |
| Total lysine | 0.77 | 0.76 |
| Total methionine + cysteine | 0.51 | 0.51 |
| Total threonine | 0.50 | 0.50 |
| Total tryptophan | 0.14 | 0.14 |
| Total valine | 0.50 | 0.50 |
| Calcium | 0.56 | 0.57 |
| Total phosphorus | 0.36 | 0.37 |

^1^ DE and ME content of the diets were calculated using energy values for the ingredients obtained from NRC (2012).

^2^ Values for SID concentrations of AA for the diets were estimated using SID coefficients for the various ingredients provided by NRC (2012).

^3^ Analyzed values are the result of a chemical analysis conducted in duplicate.
